# Sunflower Stalks versus Corn Cobs as Raw Materials for Sustainable Concrete

**DOI:** 10.3390/ma14175078

**Published:** 2021-09-05

**Authors:** Cătălina Mihaela Grădinaru, Adrian Alexandru Șerbănoiu, Bogdan Vasile Șerbănoiu

**Affiliations:** 1 Faculty of Civil Engineering and Building Services, “Gheorghe Asachi” Technical University of Iași, 700050 Iași, Romania; catalina.gradinaru@tuiasi.ro; 2 Faculty of Architecture “G.M. Cantacuzino”, “Gheorghe Asachi” Technical University of Iași, 700050 Iași, Romania; bogdan-vasile.serbanoiu@academic.tuiasi.ro

**Keywords:** vegetal aggregates, ecological concrete, natural fibers, thermal conductivity, lightweight concrete

## Abstract

Concrete, the most common material in the building industry, involves the use of mineral aggregates that represent an exhaustible resource, despite their large availability. For a series of applications, these mineral aggregates can be replaced by vegetal ones, which represent an easy renewable natural resource. In this study, two types of vegetal raw materials, namely sunflower stalks and corn cobs, were used in developing 10 compositions of ecological microconcrete, with different percentages involved: 20%, 35%, 50%, 65% and 80%; they were analyzed from the perspectives of density, compressive strength, splitting tensile strength, resistance to repeated freeze-thaw cycles, modulus of elasticity and thermal conductivity. The results revealed that the microconcretes with sunflower stalks registered slightly higher densities and better results regarding the compressive strength, splitting tensile strength, modulus of elasticity, and freeze-thaw resistance than those with corn cobs. Lightweight concrete is obtained when more than 50% replacement rates of the mineral aggregates are used.

## 1. Introduction

Buildings and construction represent high-demand sectors in terms of energy consumption and CO_2_ emissions. In 2018, they were responsible for 39% of the total CO_2_ emissions and for 36% of the global energy consumption [1]. Greenhouse gas emissions must be reduced by 50% by 2050 in order to address the global warming, according to the International Agency of Modelling [2]. In order to achieve high energy savings and emission reductions by 2050, the development of low-emission materials for this sector represent a solution to consider, besides net-zero buildings, deep renovations and low-emission energy supply [1].

Concrete represents the most used building material all over the world but it is a nonecological material by definition, if we take into account the amount of exhaustible raw materials used for its production, and the energy consumption involved in obtaining Portland cement. To produce one ton of Portland cement, around 1.5 tons of raw materials are used (limestone, marl, clay, gypsum) [2]. Cement processing involves high fuel consumption to ensure a kiln temperature of 1450 °C that results in emissions of over 500,000 tons/year of carbon monoxide, nitrogen oxide and sulfur dioxide [2], and around one ton of CO_2_ for each ton of cement produced [3].

Concrete greening methods studied so far include the substitution of cement, as its production determines an energy consumption of about 92% of the total energy [4], with various pozzolanic materials such as silica fume, metakaolin, fly ash and others [5,6,7,8,9,10]. Another greening method is the substitution of mineral aggregates into concrete composition. Although these aggregates (sand and gravel) represent a widespread natural resource worldwide, they are also exhaustible, and, in some areas, difficult or expensive to obtain. In these areas, mineral aggregates are obtained by extracting and crushing the rocks, processes that lead to negative effects on the environment, such as erosion, sandblasting, sedimentation, acid runoff, changes in relief, air pollution and noise pollution [11,12,13]. In the European Union, about 7 tons per citizen are extracted, given that the global demand is about 22 billion tons per year [14]. The extraction of aggregates is one of the most important mining industries globally, producing more than 16.5 billion tons annually [15].

Numerous studies have been carried out on replacing the mineral aggregates in concrete. They have used various variants of substitute materials for mineral aggregates, natural or industrial: bamboo [8], sugarcane [16], coconut [17], kenaf [8,18], sisal [18], hemp [8,18], expanded clay [19], pumice [19], etc. The use of aggregates with vegetal origin has advantages in terms of health [18], environmental protection [20], energy consumption [20], thermal [21] and sound [22] properties. Vegetal aggregates do not require complicated processing, contributing to pollution reduction and environment protection [23].

Among vegetal aggregates suitable for use in concrete are corn cobs and sunflower stalks, given their wide global distribution, annual regeneration nature and the encouraging results of studies carried out so far on these plants [24,25,26,27,28,29,30,31]. Corn cobs and sunflower stalks are, at the same time, agricultural waste and low-emission materials, locally available [24]. Sunflower (*Helianthus annuus*) is a widespread agricultural plant in South America, Southern Europe, European Russia and South Africa [32], grown on more than 27,000 ha globally in 2019 [33]. Maize (*Zea mays*) is a widely used agricultural plant in Eastern Europe, the United States, China, South Africa, cultivated in 2019 on more than 189,000 ha globally [34].

The quantity of corn cobs is about 15% of the total corn production. The biochemical composition of corn cobs consists of 39.1% cellulose, 42.1% hemicellulose, 9.1% lignin, 1.7% protein and 1.2% ash [35]. The main inorganic constituent is oxygen (77.52%); other constituents are silicon dioxide (10.06%), aluminum (4.44%), potassium (2.2%), calcium (2.09%), magnesium (1.49%), sodium (1.14%) and iron (1.06%). Due to the high oxygen content, corn cobs represent a good thermal insulating material, Pinto et al. [24] determining a thermal conductivity of a maize cob plate very close to that of expanded polystyrene. Their density of about 212 kg/m^3^ recommends them to be used as aggregates to obtain lightweight concrete. In a study conducted by Pinto et al. [25] on a concrete with broken corn cobs, in a ratio of 6 parts of corn cobs: 1 part of Portland cement: 1 part of water, the researchers obtained a material with a density of 382.2 kg/m^3^ and compressive strength of 120 kN/m^2^. Lightweight concrete masonry blocks with aggregates from shredded corn cobs and covered with cement paste, that were developed by Faustino et al. [30], registered a density of 1680 kg/m^3^, and maintained a high durability during the repeated freeze-thaw cycles.

The sunflower stalk represents 90% of the plant volume. The sunflower stalk consists of a compressible and light part (pith) and a peripheral woody part (bark) [31], very similar to the hemp stalk [28]. Biochemical composition for the bark is 48% cellulose and 14% lignin, and for the pith, 31.5% cellulose and 2.5% lignin [36]. The thermal conductivity of the pith is 0.039 W/m·K, a better value than glass wool (0.046 W/m·K), and that of the bark is 0.12 W/m·K. Sunflower stalk bark has a heat capacity of 1400 J/kg·K for bark, and of 1300 J/kg·K for pith, values very similar to those of hemp [36]. Its density is 105 ± 2 kg/m^3^, also very close to that of hemp, 103 ± 2 kg/m^3^ [28]. These values place the sunflower stalk among the raw materials to be used to make thermal insulation products. The sunflower stalk can be completely shredded, without the need for any specially adapted mechanism, as in the case of hemp, so its use as aggregates for ecological concrete production is easy [31]. The thermal conductivity of the aggregates obtained from the totally shredded stalk is 0.05 W/m·K, lower than that of hemp, 0.055 W/m·K [31]. In research conducted by Chabannes et al. [31] on the analysis of a concrete made with sunflower stalk aggregates as a binder, an aggregates ratio of 2, a thermal conductivity of 0.096 W/m·K and a compressive strength of about 0.5 MPa (at 60 days) were measured. Nozahic et al. [28] developed a concrete with aggregates from sunflower stalks and a binder made of limestone and pumice, with a binder/aggregate ratio equal to 18, obtaining a compressive strength at 28 days of 2.52 MPa, slightly lower than that obtained by the reference composition made with hemp instead of sunflower, 2.77 MPa.

The aim of this research is to realize a comparison between concrete with aggregates from corn cobs and concrete with aggregates from sunflower stalks. The method applied for obtaining and treating the aggregates was approached similarly to that presented in detail in a previous study [37]. A microconcrete composition with a diameter of mineral aggregates of max 8 mm was realized. In this reference composition we replaced the mineral aggregates with aggregates from sunflower stalks and then with aggregates from corn cobs at the levels of 20%, 35%, 50%, 65% and 80% by volume, resulting in 10 compositions of vegetal microconcrete. The analyses included density determination for these compositions, compressive strength, tensile strength by splitting, measurement of the modulus of elasticity, 50 repeated freeze-thaw cycles, and determination of their thermal conductivity.

## 2. Materials and Methods

The present study involved the development of microconcrete recipes with vegetal aggregates from corn cobs and sunflower stalks, respectively. As a starting point, we used a reference concrete (RC) made with a conventional microconcrete recipe of the C25/30 strength class, with a maximum aggregate size of 8 mm, according to NE 012/1-2007 [38]. This class was chosen because the mechanical strength of the concrete was expected to decrease by replacing the mineral aggregates with the vegetal ones (because lignocellulosic concretes generally have a lower strength compared to conventional ones), and the aim was to obtain an acceptable strength for the vegetal concrete compositions.

RC was made using the following materials:

- aggregates: natural sand with diameter of up to 4 mm, and river gravel with diameter of 4–8 mm;
- cement type CEM II/A-LL 42.5R MPa, produced in Romania;
- polycarboxylate ether superplasticizer additive;
- rhodanide-based accelerator additive, to speed up the early strength development of the vegetal concretes [39], so water from the composition to be used in the cement hydration process and not to be absorbed too much by the vegetal aggregates.

The water/cement ratio used was 0.50.

**Figure 1:**
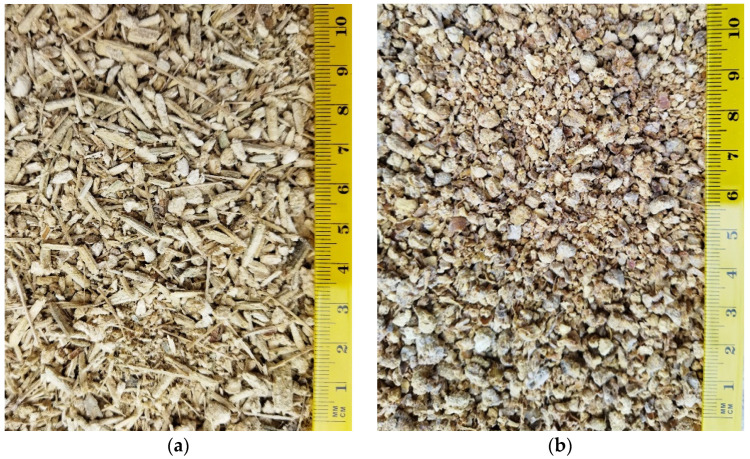
Image of vegetal aggregates: (**a**) sunflower stalks; (**b**) corn cobs.

In the RC recipe, the mineral aggregates were replaced with vegetal ones in volumetric proportions of 20%, 35%, 50%, 65% and 80%. Thus, we obtained five concrete compositions with corn cob aggregates, and five concrete compositions with sunflower aggregates. The vegetal aggregates were in granular form with variable diameter smaller than 7 mm in the case of corn cobs, and chips and fibers smaller than 25 mm length combined with granules with variable diameter of up to 7 mm in the case of sunflower stalks (Figure 1); they were obtained with the help of a mill dedicated for animal feed. After crushing, the plant aggregates were treated with 40% sodium silicate solution to reduce the water absorption capacity and to improve the interface connection with the cement matrix. After immersion into sodium silicate solution, the aggregates were left to dry on a surface heated to 52 ± 3 °C, in a room with an ambient temperature of about 24 ± 1 °C. After drying up to a constant mass, vegetal aggregate recipes were made with these aggregates. The treatment of plant aggregates is presented in more detail in a previously published study [37].

The 10 concrete recipes with vegetal aggregates were noted as SFCXX or CCCXX, XX denoting the volume percentage (20, 35, 50, 65, 80) of sunflower aggregates or corn cob aggregates, respectively.

### 2.1. The Performed Tests

In Table 1 presents in short the performed tests, including the specimens number, geometry, dimensions, and the standard applied.

### 2.2. Density Testing

The density of the hardened concrete was determined 28 days after pouring, according to the methodology of EN 12390-7:2019 [40]. The density value was obtained by calculating the arithmetic mean of three determinations.

### 2.3. Compressive Strength Testing

**Figure 2:**
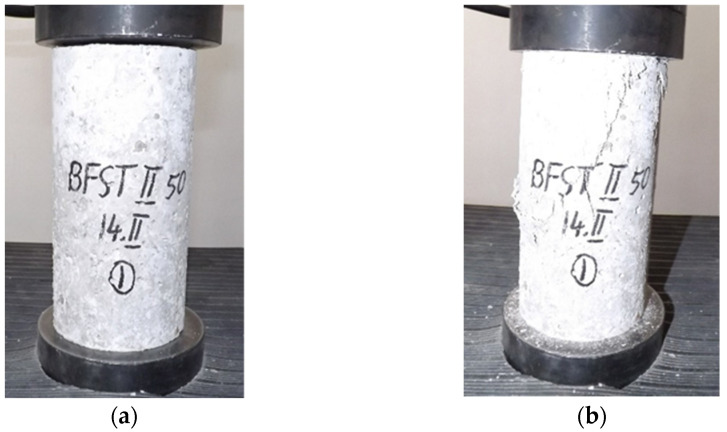
Concrete compressive strength testing. (**a**) Positioning of the test specimen; (**b**) compressive crack, after performing the test.

The concrete specimens were tested in monoaxial compression, with a hydraulic concrete press carrying a load evenly distributed on the surface of the specimen, according to EN 12390-3:2019 [41]. The specimens were cylinders with a radius of 100 mm and length of 200 mm (Figure 2). The average of three test specimens was calculated to assess the compressive strength.

### 2.4. Splitting Tensile Strength Testing

**Figure 3:**
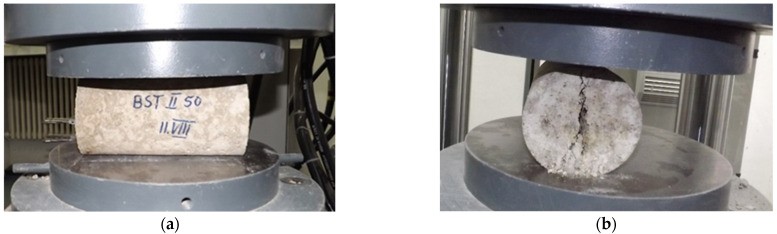
Splitting tensile concrete testing. (**a**) positioning of the test specimen, longitudinal view; (**b**) splitting crack, after performing the test.

The determination of splitting tensile strength was performed on cylinders with diameter of 100 mm and length of 200 mm. This test consisted in compressing a specimen after two diametrically opposed generators (Figure 3).

The tests were performed according to EN 12390-6:2009 [42]. A hydraulic press was used to perform the tests, recording the value of the breaking force. The average of the results of three tests for each concrete composition was calculated.

### 2.5. Freeze-Thaw Resistance Testing

**Figure 4:**
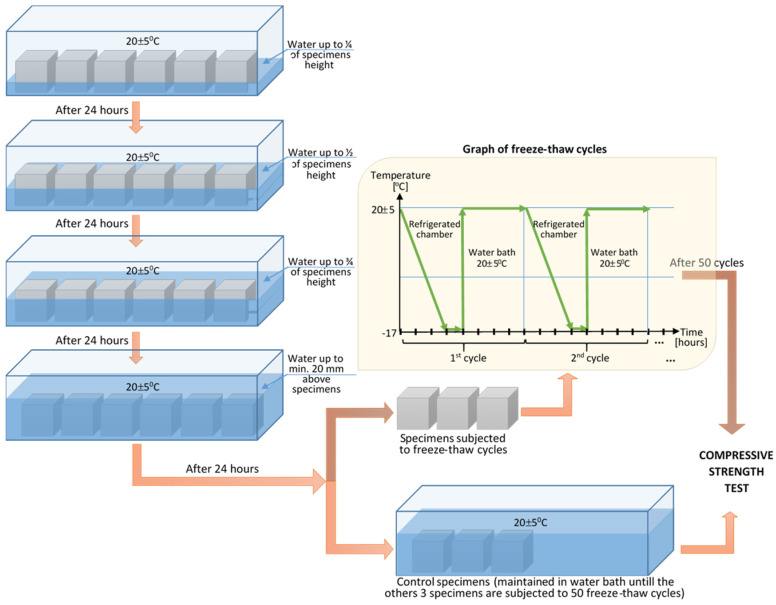
Method applied for freeze-thaw resistance determination.

**Figure 5:**
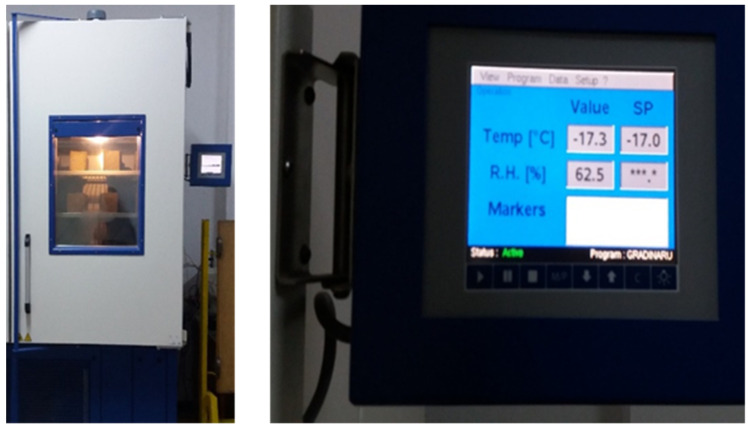
Refrigerated chamber used for freezing the concrete specimens.

The freeze-thaw resistance test (Figure 4) was performed according to SR 3518:2009 [43], regarding the determination of the freeze-thaw resistance, by measuring the variation of the compressive strength. To perform this test, cube-type specimens with sides of 100 mm were made, and the test was performed after the concrete age of 28 days. The concretes were subjected to 50 freeze-thaw cycles. Six specimens were made for each concrete composition, three of them serving as control specimens and the other three being subjected to freeze-thaw cycles. The operation method required that all concrete specimens be placed in a water bath at a temperature of 20 ± 5 °C, four days before the start of the actual test. The water level was initially one quarter of the height of the specimen; after 24 h it rose to one half the height of the specimens, and then three-quarters, and after another 24 h it rose at least 20 mm above the specimens. The control specimens always remained in the water bath during the freeze-thaw test, and those to be tested for freezing and thawing were placed in a cold room at a constant temperature of −17 ± 2 °C (Figure 5) for at least 30 min at the end of the frost stage. After 4 h, they were removed from the cold room and placed in a water bath at 20 ± 5 °C.

After completing 50 cycles of 4 h of freezing and 4 h of thawing, the specimens were compression tested, and the loss of compressive strength was determined.

### 2.6. Modulus of Elasticity Testing

**Figure 6:**
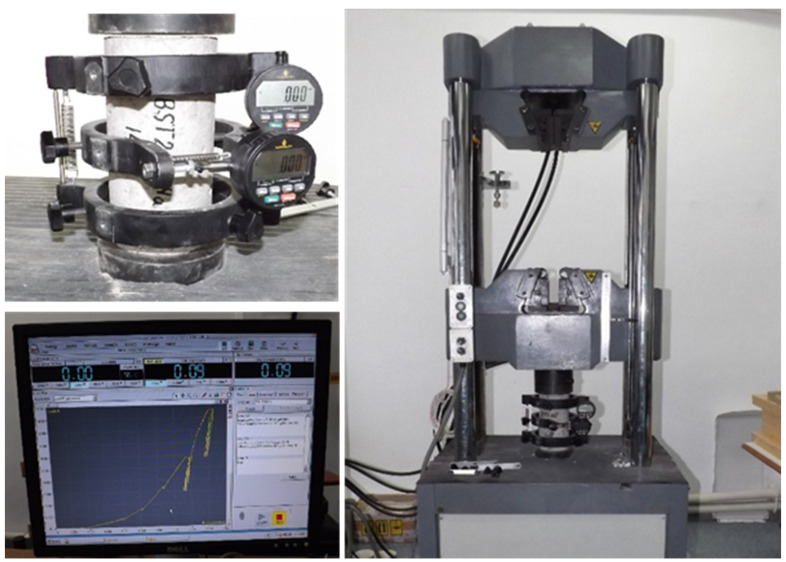
Equipment used to measure displacement in determining the modulus of elasticity of concrete.

For determination of the modulus of elasticity to compression of the concrete developed in this research, method 2 of EN 13412:2006 [44] was applied, adequate for products based on cement binders. The equipment used to perform these determinations was a 100 ton hydraulic press and a compressometer/extensometer for cylinders with digital indicator, according to Figure 6.

The modulus of elasticity is determined by measuring the change in tension in the concrete test specimen at loading, to produce a pressure (force) between 0.5 N/mm^2^ and one third of the compressive strength of the specimen, determined according to EN 12390-3:2019 [41].

### 2.7. Thermal Conductivity Testing

The thermal conductivity was determined according to a method provided in SR EN 12667:2009 [45] on the thermal performance of construction materials and products, namely the thermoflowmeter method, and according to C155:2013 [46].

## 3. Results and Discussions

**Figure 7:**
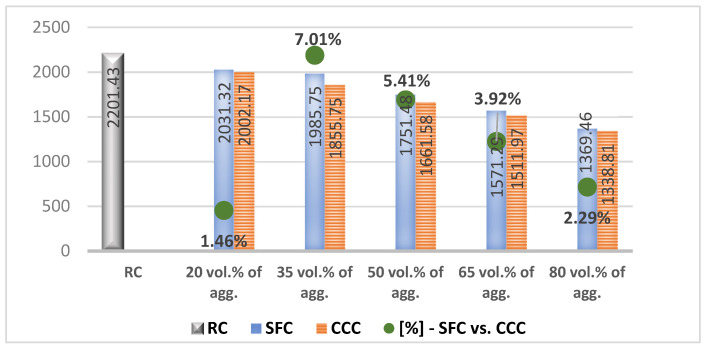
Apparent density of concrete with 20, 35, 50, 65 and 80% vegetal aggregates, measured 28 days after pouring [kg/m^3^].

**Figure 8:**
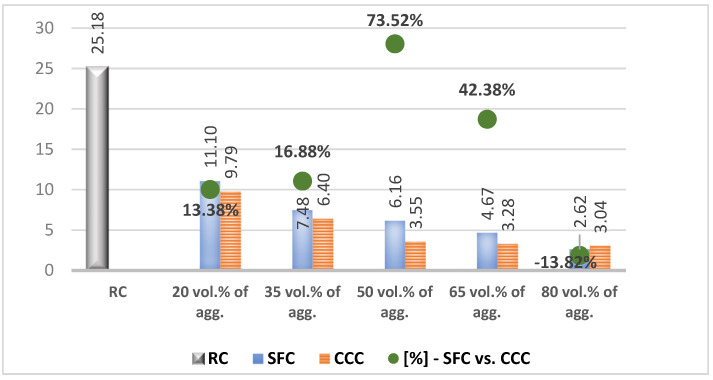
Compressive strength of concrete with 20, 35, 50, 65 and 80% vegetal aggregates, determined 28 days after pouring [N/mm^2^].

**Figure 9:**
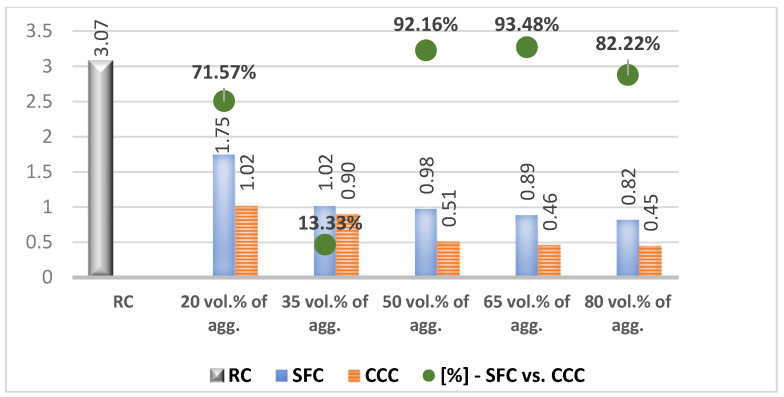
Splitting tensile strength of the concretes with vegetal aggregates [N/mm^2^].

**Figure 10:**
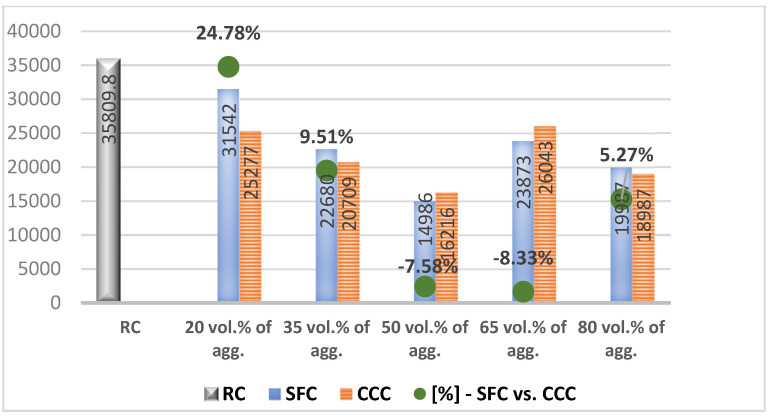
Modulus of elasticity of concrete with vegetal aggregates.

**Figure 11:**
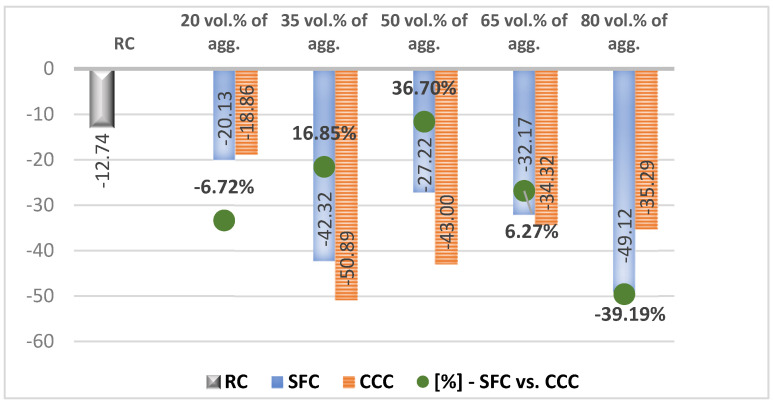
Loss in compressive strength of concrete with aggregates from sunflower stalks versus that with aggregates from corn cobs, following freeze-thaw testing [%].

**Figure 12:**
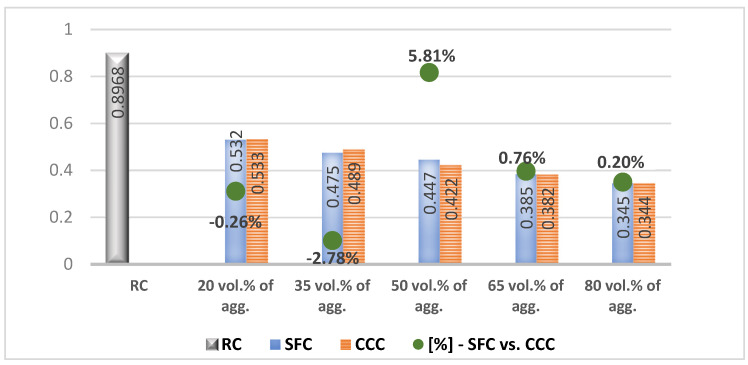
Thermal conductivity of concrete with sunflower aggregates versus that with corn cob aggregates [W/m·K].

In the results analysis, we realized an interpretation of the individual values of the vegetal concretes (the columns from the Figure 7, Figure 8, Figure 9, Figure 10, Figure 11 and Figure 12) comparative to the RC value written in the beginning of each test discussion, and one of the values obtained by SFC comparative to those of CCC (the green dots from the Figure 7, Figure 8, Figure 9, Figure 10, Figure 11 and Figure 12). When these green-dot values are positive, they denote how much the SFC value is above the CCC value. When the green dots have negative values, this means how much the SFC value is below the CCC value. The comparison was performed for each replacement rate group, so SFC20 was compared to CCC20, SFC35 to CCC35, and so on.

### 3.1. Density

RC registered a density of 2201.43 kg/m^3^. From Figure 7 analysis, it can be seen that the density of vegetal concretes decreases as the rate of plant material increases. The replacement of 20% of the mineral aggregates with those of corn cobs led to a reduction in the concrete density by 9%, while the replacement by 35%, 50%, 65% and 80% resulted in a density of 15.70%, 24.52%, 31.32% and 39.18% lower, respectively, compared to RC density. These variants decrease in density by between 6–8% from one variant to another. Replacing 20% of mineral aggregates with aggregates from sunflower stalks reduced the density of the concrete by approx. 8%, while their replacement in rates of 35%, 50%, 65% and 80% resulted in decreases in the concrete density by 15.70%, 20.62%, 28.72% and 37.97%, respectively, compared to RC. The density of the vegetal concrete decreased by 6–9% from one compositional variant to another, in the case of SFC20, SFC35, SFC50, SFC65 and SFC80.

The density of concrete with corn cobs, respectively with sunflower stalks, measured 28 days after pouring, decreased as the share of plant material in the composition increased. Analyzing comparatively the density of concrete with sunflower aggregates and that with corn cob aggregates (Figure 7, the green points) we observed slightly higher densities in the case of the first type of aggregates, by 1.3–5.2% in the case of replacing mineral aggregates with up to 80% vegetable aggregates. Lightweight concrete is obtained with a percentage of more than 50% of plant matter. In general, concrete compositions made with sunflower aggregates had higher densities than those with aggregates from corn cobs. This is due to the organic chemical composition of plant aggregates. Thus, although aggregates from sunflower stalks have a lower bulk density than those from corn cobs, the majority of cellulose in their structure causes a higher absorption of cement paste than in the case of corn cobs, thus causing a more compact packaging and a higher density per unit volume of vegetal concrete. The majority of lignin content in the case of corn cob aggregates results in a lower absorption capacity, and thus obtains a lighter vegetal concrete, despite the higher bulk density of corn cobs than the sunflower stalks. It can be concluded that the most important role in obtaining a lighter concrete belongs to the internal structure of the used plant aggregates, namely, their absorption capacity of the cement paste.

According to [45], lightweight concrete aggregates can be classified according to their density in the following classes: D0.8; D1.0; D1.2; D1.4; D1.6; D1.8 and D2.0. Table 2 presents these density classes, and the distribution of the developed concrete compositions. As CCC20 and SFC20 registered a density over 2000 kg/m^3^, they are not lightweight concretes and were not included in this table.

In a comparative analysis with other studies results, the densities obtained in this study are below of those obtained by [30], which used similar shredded corn cob aggregates but covered with cement paste, as 15% replacement of the mineral ones. The material obtained with 80% corn cob aggregates recorded a density 3.5 times higher than the material developed by [25] with corn cobs broken into pieces.

### 3.2. Compressive Strength

RC registered a compressive strength of 25.18 N/mm^2^. From the Figure 8 analysis, it can be seen that as the share of plant aggregates increased, the compressive strength of the obtained concrete decreased. The replacement of mineral aggregates with 20% corn cob aggregates diminished the RC compressive strength by approx. 60%, registering a value of 9.8%. The increase by 15% of the share of corn cob aggregates determined the decrease by approx. 35% of the compressive strength in the case of CCC35, and the next stage of increasing this percentage by another 15% determined the decrease of the compressive strength of the concrete by approx. 45% compared to the previous version. In the case of CCC65 and CCC80 compositions, although the share of plant matter continued to increase with same percentage, the compressive strength decreased by approx. 7.5% compared to the previous variant.

According to Figure 8, the replacement of 20% of the mineral aggregates with sunflower aggregates determined a decrease of the RC compressive strength by approx. 56%, and in the case of 35% replacement of their volume, a decrease of another 30% compared to the previous version. Therefore, a surplus of plant sunflower aggregates of 15% resulted in a double decrease in compressive strength (30%). The further increase of the share of sunflower aggregates, from 35% to 50%, determined the decrease of the compressive strength by approx. 76% compared to RC, but with only 18% compared to the previous version. From the level of 50%, the rate of decrease of the compressive strength started to increase from one variant to another, as the rate of the vegetal material in the total volume of the concrete increased by 15%. Thus, in the case of SFC65, the compressive strength decreased by 25% compared to SFC50, and in the case of SFC80, by 44% compared to the previous version. Compared to RC, the replacement of mineral aggregates in a proportion of more than 65% resulted in a decrease in compressive strength by more than 80%, below 5 N/mm^2^, with SFC80 reaching 2.62 N/mm^2^.

Comparatively analyzing the values obtained by the concrete with sunflower aggregates vs. concrete with corn cob aggregates from Figure 8, the green points, it can be seen that the sunflower aggregates determined higher compressive strength. Thus, in case of replacing the mineral aggregates with 20% and 35% vegetal aggregates, concrete with sunflower obtained higher compressive strengths by approx. 10% and 16%, respectively, than the concrete with corn cobs. In the case of replacing 50% of the mineral aggregates, the difference between the two variants was over 70% in favor of concrete with sunflower aggregates. The explanation for these significant improvements in SFC versus CCC is related to the absorption capacity of cement paste of sunflower aggregates, and in the case of SFC50 and CCC50, an additional contribution was the more efficient packaging of plant aggregates—mineral aggregates, simultaneously with the higher absorption capacity of the sunflower aggregates.

In a comparative analysis with other studies’ results, the material obtained with 80% corn cob aggregates recorded a compressive strength 22 times higher than the material developed by [25] with corn cobs broken into pieces. The compressive strength of SFC80 is similar to the one recorded by the concrete developed by [28] and 5.2 times higher than the one of the material studied by [31].

### 3.3. Splitting Tensile Strength

RC registered a splitting tensile strength measured at 28 days of 3.07 N/mm^2^. In Figure 9 it can be seen that the splitting tensile strength of the corn cob concretes decreased as the plant material increased. Thus, the use of 20% aggregates from corn cobs resulted in a loss of this type of resistance of over 66%. The increase of the share of corn cob aggregates by 15% determined further the decrease of this parameter, but to a much smaller extent, by approx. 12% compared to the previous version. The same situation was seen in the case of increasing the share from 50% to 65%. In the case of CCC50, the increase of the corn cobs’ share compared to the previous variant determined the decrease of the splitting tensile strength by around 43%. The increase of the corn cob percentage from 65% to 80%, determined an improvement of the splitting tensile strength by approx. 3%. The best splitting tensile strength between concretes with corn cob aggregates was recorded by CCC20.

In the case of concrete with sunflower aggregates, there was also a decrease in splitting tensile strength as the percentage of plant material increased (Figure 9). The increase from 20% to 35% and from 50% to 65% determined the decrease of this parameter by 44.51%, and 18.64%, respectively, compared to the lower variant. On the other hand, the increase of this proportion from 35% to 50%, and from 65% to 80%, determined the improvement of the splitting tensile strength by 3.34% and 7.78%, respectively, compared to the inferior variant. The best value of splitting tensile strength among the concretes with sunflower aggregates was recorded by SFC20 (1.72 N/mm^2^); the following variants, SFC35, SFC50, SFC65, and SFC80, recorded approximately similar values of this parameter, with small variations between 0.80–0.99 N/mm^2^.

In a comparative analysis, according to Figure 9, the concrete with sunflower aggregates registered a better splitting tensile strength than that with corn cob aggregates. The strength difference between the two concrete variants was about 70% in the case of 20% replacement of mineral aggregates with the vegetal ones, 80% in the case of replacement of 65% of their volume, and of around 82% in the case of 80% replacement. The use of 50% vegetal aggregates determined a higher value of the splitting tensile strength by about 94% in the case of concrete with sunflower aggregates, compared to the one with corn cob aggregates.

### 3.4. Modulus of Elasticity

RC registered a modulus of elasticity of 35,809.80. According to Figure 10, the modulus of elasticity of concrete compositions with sunflower stalk aggregates decreases as the share of plant material increases, this proving a more elastic character under the action of compressive forces. The same effect was obtained in the case of concrete with aggregates from corn cobs. Analyzing comparatively, according to Figure 10, the green points, SFC registered higher values of this parameter than CCC when the 20, 35 and 80 replacement rates were applied.

### 3.5. Freeze-Thaw Resistance

In terms of compressive strength, after being subjected to 50 freeze-thaw cycles, RC registered a decrease of 12.74%. Concrete with corn cob aggregates recorded decreases to a greater extent than RC (Figure 11). Thus, given that the RC resistance decreased by 12.74%, in the case of CCC20 this resistance decreased by approx. 19%, and in the case of CCC65 and CCC80, a decrease of around 35%. In the case of CCC50, the decrease in compressive strength was 43%, and in the case of CCC35, around 51%. In the case of concrete with sunflower aggregates, the 50 freeze-thaw cycles also led to a decrease in its compressive strength.

In comparative analysis (Figure 11, the green points), concrete with sunflower aggregates obtained better results than concrete with corn cob aggregates when using a substitution rate of 35% and 50% of mineral aggregates with vegetal aggregates, while in the case of 20% and 80% substitution rates, we recorded compressive strengths lower by 11.90% and 44.60%, respectively, than the one of concrete with corn cob aggregates. When 65% of the mineral aggregates were replaced with vegetal ones, the loss of compressive strength was almost similar for both types of vegetal aggregates.

### 3.6. Thermal Conductivity

RC registered a thermal conductivity of 0.8968 W/m·K. According to Figure 12, it can be observed that as the substitution rate of the vegetal aggregates into the concrete composition increases, its thermal conductivity decreases.

Thus, by using a substitution rate of 50% of mineral aggregates with corn cob aggregates, the thermal conductivity of the RC decreased by about 53%, registering a value of 0.4220 W/m·K. If the corn cob aggregates’ share increased by 15% from one concrete variant to another, until the level of 80%, the thermal conductivity decreased between 8.30% and 13.63%, respectively. Thus, CCC35 registered a thermal conductivity lower by 8.30% than CCC20, CCC50 registered a thermal conductivity lower by 13.63% than CCC35, CCC65, by 9.50% than CCC50, and CCC80, by 9.94% compared to CCC65.

From Figure 12 analysis, the 50% substitution of the mineral aggregates with sunflower aggregates led to a decrease of the thermal conductivity of the RC by 50.21%, registering the value of 0.4465 W/m·K. The increase by 15% (up to 80%) of the share of sunflower aggregates in the concrete composition determined decreases of its thermal conductivity by 6–14% from one compositional variant to another. Thus, SFC35 and SFC80 registered a lower thermal conductivity by about 10.5% compared to SFC20 and SFC65, respectively. SFC50 recorded a decrease in thermal conductivity by 6% than SFC35, and SFC65, by about 14% compared to SFC50.

In a comparative analysis (Figure 12, the green points), the concrete with sunflower aggregates registered a lower thermal conductivity than the concrete with corn cob aggregates, by 0.27% in the case of the 20% substitution rate, and by 2.78% for the 35% substitution rate. In the case of using 65% and 80% replacement rates, the difference between the two types of aggregates was insignificant, and in the case of a 50% replacement of mineral aggregates, the concrete with sunflower aggregates recorded a thermal conductivity 5.81% higher than the concrete with corn cob aggregates.

Comparative to the results of other studies, SFC80 recorded a 3.5 times smaller thermal conductivity than that obtained by [31].

## 4. Conclusions

This research aimed to provide a comparative analysis between microconcrete compositions with aggregates from sunflower stalks and microconcrete compositions with aggregates from corn cobs. The results obtained led to the formulation of the following conclusions:

- The vegetal aggregates led to concrete density decreasing, as their content increased. As a reference example, the replacement of 50% of mineral aggregates led to around 20% lower density than RC in the case of SFC, and with around 24% in the case of CCC. Lightweight concretes were obtained in the case of mineral aggregates replacing in percentages greater than or equal to 50%, no matter the vegetal raw material used, sunflower stalks or corn cobs. Corn cob aggregates led to lower densities of the microconcretes than those of sunflower stalks.
- The compressive strength of the RC was decreased by the vegetal aggregates. The replacement rate of 50%, for example, led to a 75% decrease for SFC and 86% for CCC case. Sunflower stalk aggregates determined higher compressive strengths of the microconcretes than those of corn cobs; the microconcretes made with 50% or smaller rates of sunflower aggregates and those with 35% or smaller rates of corn cob aggregates can be used in nonstructural applications since they registered compressive strengths higher than 6 N/mm^2^.
- As regards the splitting tensile strength, compared to RC, vegetal concretes registered also smaller values, SFC50 for example, with 68%, and CCC50 with 83%. The compositions with sunflower aggregates led to significantly higher values than those with corn cob aggregates due to the difference between the two types of vegetal aggregates, the sunflower aggregates being in the form of chips and fibers also, and not just as rounded granules as in the case of corn cob aggregates.
- From the analysis of elasticity, the concrete compositions with up to 35% vegetal matter showed a higher modulus of elasticity in the case of using aggregates from sunflower stalks; for replacement rates of mineral aggregates higher than 35%, aggregates from corn cobs determined higher values of this parameter; compared to RC, the vegetal concretes registered lower values, so they presented higher elastic behavior.
- The vegetal aggregates led to a diminished resistance to repeated freeze-thaw cycles, a rate of 50% in the concrete composition determined a 14% bigger decrease than RC in case of SFC, and one of 30% in case of CCC. Aggregates from sunflower stalks recorded better results than those from corn cobs when using a substitution rate of mineral aggregates between 35% and 65%.
- The thermal conductivity of the RC was very much reduced by the vegetal aggregates. The two types of plant aggregates led to relatively similar results, the differences being very small; the use of a 50% replacement rate of mineral aggregates with vegetal aggregates from sunflower stalks or corn cobs led to a decrease of about 50% in the thermal conductivity of RC; thus, these vegetal aggregates determined an important improvement of the insulation properties of the ordinary concrete.

In a comparative analysis with other studies results, CCC recorded densities below those that implied other treatment of the vegetal aggregates (namely, cement paste covering), and above those that implied broken corn cobs, not shredded. The compressive strength of CCC80 was above other studies on concrete with similar corn cob content. SFC80 recorded bigger or similar values for compressive strength and better thermal conductivity than similar studied materials.

As for advantages of sunflower stalks and corn cobs in concrete composition, they had a significant influence in reducing density and thermal conductivity, in addition to their ecological and sustainable character. A concrete with lower density and a more elastic character could lead to a better behavior of the buildings in case of seismic activity, since it would lower overall weight if it is used in nonstructural applications, such as screeds, plaster, partition walls or in masonry blocks form. As screeds, adding their thermal insulation characteristics, a reduction of the thermal bridges at the slab level can be obtained.

SFC and CCC can improve a building’s sustainability by diminishing their total carbon footprint through the use of easy-to-obtain, renewable raw materials instead of exhaustible ones.

## Figures and Tables

**Table 1:** Performed tests summary.

Test	Specimens Number Tested for Each Composition	Specimen Geometry	Specimen Dimensions	Standard Applied
Density	3	cylinder	100 mm radius 200 mm length	EN 12390-7:2019 [39]
Compressive strength	3	cylinder	100 mm radius 200 mm length	EN 12390-3:2019 [40]
Splitting tensile strength	3	cylinder	100 mm radius 200 mm length	EN 12390-6:2009 [41]
Freeze—thaw resistance	6	cube	100 mm	SR 3518:2009 [42]
Modulus of elasticity	3	cylinder	100 mm radius 200 mm length	EN 13412:2006 [43]
Thermal conductivity	3	cuboid	300 × 300 × 50 mm	SR EN 12667:2009 [44] C155:2013 [45]

**Table 2:** Classification of lightweight concretes developed in this research by density classes.

Density Mass Class [45]	Density Mass Range [45] [kg/m^3^]	Vegetal Concrete Compositions
D1,4	(1200, 1400]	CCC80, SFC80
D1,6	(1400, 1600]	CCC65, SFC65
D1,8	(1600, 1800]	CCC50, SFC50
D2,0	(1800, 2000]	CCC35, SFC35

## Data Availability

Data is contained within the article and in [37] for the method of vegetal aggregates obtaining.
